# A Predictive Phosphorylation Signature of Lung Cancer

**DOI:** 10.1371/journal.pone.0007994

**Published:** 2009-11-25

**Authors:** Chang-Jiun Wu, Tianxi Cai, Klarisa Rikova, David Merberg, Simon Kasif, Martin Steffen

**Affiliations:** 1 Department of Biomedical Engineering, Boston University, Boston, Massachusetts, United States of America; 2 Department of Biostatistics, Harvard School of Public Health, Boston, Massachusetts, United States of America; 3 Cell Signaling Technology, Danvers, Massachusetts, United States of America; 4 Vertex Pharmaceuticals, Cambridge, Massachusetts, United States of America; 5 Children's Hospital Informatics Program at the Harvard-MIT Division of Health Sciences and Technology, Boston, Massachusetts, United States of America; 6 Center for Advanced Genomic Technology, Boston University, Boston, Massachusetts, United States of America; 7 Department of Pathology and Laboratory Medicine, Boston University School of Medicine, Boston, Massachusetts, United States of America; Baylor College of Medicine, United States of America

## Abstract

**Background:**

Aberrant activation of signaling pathways drives many of the fundamental biological processes that accompany tumor initiation and progression. Inappropriate phosphorylation of intermediates in these signaling pathways are a frequently observed molecular lesion that accompanies the undesirable activation or repression of pro- and anti-oncogenic pathways. Therefore, methods which directly query signaling pathway activation via phosphorylation assays in individual cancer biopsies are expected to provide important insights into the molecular “logic” that distinguishes cancer and normal tissue on one hand, and enables personalized intervention strategies on the other.

**Results:**

We first document the largest available set of tyrosine phosphorylation sites that are, individually, differentially phosphorylated in lung cancer, thus providing an immediate set of drug targets. Next, we develop a novel computational methodology to identify pathways whose phosphorylation activity is strongly correlated with the lung cancer phenotype. Finally, we demonstrate the feasibility of classifying lung cancers based on multi-variate phosphorylation signatures.

**Conclusions:**

Highly predictive and biologically transparent phosphorylation signatures of lung cancer provide evidence for the existence of a robust set of phosphorylation mechanisms (captured by the signatures) present in the majority of lung cancers, and that reliably distinguish each lung cancer from normal. This approach should improve our understanding of cancer and help guide its treatment, since the phosphorylation signatures highlight proteins and pathways whose phosphorylation should be inhibited in order to prevent unregulated proliferation.

## Introduction

At the molecular level, cancers are heterogeneous diseases, arising from genetic factors, environmental carcinogens and random, somatic mutation [1]. Phosphorylation of proteins is a key regulator of protein activity [2], and in particular, modification of tyrosine residues modulate critical signaling and control processes [3]. In cancers, aberrant phosphorylation status of key residues (its presence or absence) has been observed and documented in many studies, which include the original oncogene, src [4], and many others [5].

Signatures based on protein levels are starting to be developed [6]. Protein levels are expected to be strongly correlated with phenotype and protein-based diagnostics can be easily implemented in most major medical centers. Monitoring the functional status of proteins may therefore be highly germane to clinical applications, and offer an additional layer of specificity for enhancing our scientific understanding of causal progression of disease. Methods for high-throughput detection of phosphorylated residues using mass spectrometry are being rapidly developed [7], [8], [9], [10] and applied to the study of signaling pathways [11] along with complementary analysis and modeling approaches [12], [13].

In this paper, we examined global tyrosine phosphorylation data from lung cancers and normal lung tissue [14], seeking to identify differentially phosphorylated protein sites and differentially activated pathways, and to assess their suitability as classifiers. We report a large set of sites that are differentially phosphorylated in tumors, many of which can be used as direct targets for new drugs. We present evidence that certain pathways are differentially activated, based on their global phosphorylation status using a novel computational approach to perform a protein variant of gene set enrichment analysis.

We then demonstrate that a relatively small number of phosphorylated peptides observed in that data [14] can discriminate between normal tissue and tumor with exquisite sensitivity and specificity. We validate our phosphorylation signature using rigorous cross validation and testing on a previously unpublished independent set. Finally, we compare the binding affinities of multiple kinase inhibitors with the phosphorylation activity of their targets in our study. The integration with pharmaceutical data leads to interesting hypotheses about the relative efficacy of such drugs and suggests unexplored but potentially potent lung cancer agents, highlighting potential clinical applications.

There is a fundamental distinction between predictive signatures, such as the ones developed here, and the observation that a protein is differentially expressed (or phosphorylated) with statistical significance. In principle, a protein may be differentially phosphorylated but be of little predictive utility for the broad classification of a disease or for devising a personalized treatment strategy. Differential phosphorylation of a protein is a population aggregate summary. It means that, on average, the phosphorylation level of a protein is higher or lower in a cancer than normal tissue. However, for any given patient the probability of error in classifying the biopsy as a cancer could be as high as 0.49 (if the distributions of the measurements for cancer and normal tissues overlap). No account of disease heterogeneity is considered, and elevated levels could result solely from a subset of the disease cases. Conversely, a signature with high predictive value suggests that the phospho-sites included in the signature are part of a core set of pathways that are universally operative in the disease. They are therefore potentially reflective of a universal pathogenetic mechanism for that disease, and can lead to the discovery of a “phosphorylation logic” that captures the tissue specific, or even a general, neoplastic phenotype. Heterogeneous cancer sub-types will necessarily require more complex signatures, requiring a large set of predictive mechanisms that can provide high-coverage of the differential activity of key pathways in the specific cancer. Finally, if the predictive signatures consist of a small set of proteins that implicate specific pathways (as implied by our work), this set of pathways becomes a prime target for a broad combinatorial multi-target drug strategy.

## Results

### Multiple Tyrosine Sites Are Differentially Phosphorylated in Lung Cancer Tissue

We first analyzed individual protein sites to determine those that are differentially phosphorylated between the 48 normal and 94 non-small cell lung cancer (NSCLC) tumor samples. Our results reveal 129 unique amino acid sites that were significantly differentially phosphorylated between normal and tumor samples (false discovery rate, FDR *q* value <0.05). Of these, 77 of the sites were more phosphorylated in cancerous tissue and 52 sites were more phosphorylated in normal tissue. Table 1 lists the highest-ranking 20 protein sites with the smallest ranksum *p* values, with all sites listed in Table S1.

**Table 1:** The 20 protein sites most differentially phosphorylated between normal and NSCLC samples.

Index	ID	T/N SCR*	*P*-value**	FDR***	Description
1	ADH1B_34	0.08	5.13E-12	1.35E-09	Alcohol dehydrogenase IB (class I), beta polypeptide
2	CAV1_14	0.15	2.13E-11	2.27E-09	caveolin 1, caveolae protein, 22kDa
3	TNS1_1149	0.16	2.58E-11	2.27E-09	tensin 1
4	C11ORF52_103	0.13	4.32E-11	2.85E-09	chromosome 11 open reading frame 52
5	GAB1_659	0.17	3.26E-10	1.72E-08	GRB2-associated binding protein 1
6	TNS1_1326	0.19	8.84E-10	3.89E-08	tensin 1
7	ANXA2_29	0.2	4.40E-09	1.66E-07	Annexin A2
8	TNS1_1404	0.13	1.12E-08	3.62E-07	tensin 1
9	STAT1_701	0.05	1.23E-08	3.62E-07	signal transducer and activator of transcription 1, 91kDa
10	LYN;HCK_396;410	3.87	3.46E-08	9.14E-07	v-yes-1 Yamaguchi sarcoma viral related oncogene homolog //// hemopoietic cell kinase
11	CDC2_15	9.36	7.75E-08	1.86E-06	cell division cycle 2, G1 to S and G2 to M
12	CDC2_15,19	13.45	1.25E-07	2.75E-06	cell division cycle 2, G1 to S and G2 to M
13	C19ORF59_38	0.08	1.40E-07	2.84E-06	chromosome 19 open reading frame 59
14	SEPT2_17	0.08	1.71E-07	3.23E-06	septin 2
15	TNS1_1323	0.14	1.86E-07	3.27E-06	tensin 1
16	C11ORF52_78	0.09	2.06E-07	3.41E-06	chromosome 11 open reading frame 52
17	TJP2_1118	0.25	3.55E-07	5.51E-06	tight junction protein 2 (zona occludens 2)
18	PTTG1IP_174	4.03	8.59E-07	1.26E-05	pituitary tumor-transforming 1 interacting protein
19	MAPK13_182	2.72	1.03E-06	1.39E-05	mitogen-activated protein kinase 13
20	PIK3R2_464	4.87	1.05E-06	1.39E-05	phosphoinositide-3-kinase, regulatory subunit 2 (p85 beta)

* T/N SCR: Tumor/normal phosphorylation spectral count ratio;

** P-value: significance of difference between two sample groups with rank sum test;

*** FDR: False discovery rate correction of the p values.

In addition to the top genes listed in the table many other prominent markers of cancer were detected in our analysis. In particular, EGFR is a receptor tyrosine kinase implicated in lung cancer and is involved in multiple biological processes, including apoptosis, cell adhesion, and growth [15], [16], [17]. Mutations of EGFR are seen in a set of NSCLC patients with good response to EGFR inhibitor [18], [19]. The phosphorylation statuses of two tyrosines on the cytoplasmic tail of EGFR were found to be statistically different, exhibiting greater levels of phosphorylation in cancer. The residues Y1172 and Y1197, are known to regulate proliferative activity [20]. Interestingly, Y1172 is hyper-phosphorylated (Tumor/Normal phosphorylation spectral count ratio >1) only in adenocarcinoma (AD) samples. Y1197 is hyper-phosphorylated in both AD and squamous cell carcinoma (SCC) subtypes, but to a significantly higher degree in AD.

The site specific analysis revealed, somewhat surprisingly, that the amino acid most consistently differentially phosphorylated in normal and tumor tissue was Y34 of alcohol dehydrogenase 1B, ADH1B ( less phosphorylated in tumors). This protein participates in multiple related processes such as glycolysis, gluconeogenesis, and fatty acid metabolism. Its expression is up-regulated in the late stage of rat lung development, but down-regulated in human NSCLC [21]. The specific role of Y34 is currently unknown, but the low phosphorylation count may reflect either compromised enzyme activity or the decreased protein abundance in tumors. With its role in alcohol metabolism, this may be a consequence of the Warburg effect whereby tumors employ aerobic glycolysis to meet their metabolic needs [22]. ADH1B was recently identified as a risk modifier for squamous aerodigestive cancers, with a postulated mechanism of altered ethanol metabolism as being contributory [23]. Another study noted decreased protein levels of ADH1B in breast tumors [24], postulating the inability to oxidize the hydroxyl group of retinol blocks the production of retinoic acid, a molecule that helps maintain epithelial cells in their differentiated state.

There were many other differentially phosphorylated proteins. Those hyperphosphorylated in tumors include multiple receptor tyrosine kinases (listed in Table S2), and other signaling proteins, such as p38 delta, protein kinase C delta, and members of the PI3K signaling pathway, including p85 beta. Conversely, proteins hypophosphorylated (Tumor/Normal phosphorylation spectral count ratio >1) in tumors include the transcription factors STAT1 and STAT5, the protein tyrosine phosphatase PTPN11, the G-protein coupled receptor GPRC5A, and the kinases MAPK1, MAPK3, and TNK2.

### Highly Accurate Classification of Tumor Tissue via Cross Validation

In order to assess the potential utility of monitoring protein activity via tyrosine phosphorylation data, we developed classifiers to predict the cancer/normal phenotypes of individual samples. In particular, we examined our ability to distinguish normal tissue from cancers based on small set of phosphotyrosines. Table 2 summarizes the performances of five predictive models we examined. Two of the models were based on statistically selected sites that yield maximum discriminating power between cancer and normal tissue. Three models were based on biologically driven selection of sites from key pathways associated with lung cancer. A regularized regression model (aiming to reduce the likelihood of overfitting the data) based on all significantly differentially phosphorylated protein sites (FDR *q* value <0.05) successfully predicted the sample classes with an average classification accuracy of 0.925 and an area-under-the-curve, AUC at 0.974 in a rigorous bootstrapped cross validation analysis that carefully separates training on random subsets of the data and testing on the remaining subset. The selection of most informative sites used to construct the model was also done on training data. The average number of phosphotyrosine sites used across all bootstrap trials was 88.

**Table 2:** The performances of the predictive models for normal/tumor classification.

Marker Sites Used in the Regression Models	Classification Accuracy (95% C.I.)	AUC (95% C.I.)	Average No. of Marker Sites in the Model
Differentially phosphorylated sites	0.925	0.974	88
	(0.833∼0.986)	(0.925∼1.000)	
Proliferation category	0.81	0.912	17
	(0.712∼0.859)	(0.858∼0.945)	
EGFR pathway from BioCarta	0.764	0.826	12
	(0.637∼0.85)	(0.718∼0.894)	
EGFR signaling network from HPRD	0.887	0.957	47
	(0.791∼0.961)	(0.892∼0.991)	
Top 20 sites	0.883	0.944	20
	(0.757∼0.962)	(0.820∼0.995)	

Shown in the table are the mean classification accuracy and AUC across the 100 bootstraps. The 95% bootstrap confidence intervals (C.I) of the accuracy and AUC are in the parentheses.

We also investigated whether biologically informed models based on relevant gene modules can deliver an equal accuracy. Specifically, regression models based on genes in the MSigDB “Proliferation” protein-set (commonly referred to as “gene sets” in the microarray literature) [25], and two different protein-sets representing the EGFR pathway, were also shown to discriminate between normal and tumor samples with high accuracy. For the EGFR pathway, we considered two representations: a “core” pathway with 11 proteins (Biocarta) and an “expanded” pathway with 47 proteins (HPRD). The proteins are listed in Table S3. Our results suggest that while the core EGFR proteins do provide a reasonable accuracy in distinguishing cancer versus normal tissue (0.83 AUC), the “expanded” EGFR pathway, with additional proteins, performs significantly better (0.96 AUC). The most informative proteins in the expanded pathway not included in the core EGFR network are CAV1, GAB1, PXN, and PTPN11. Lastly, a model based on the top 20 performing sites has an average classification accuracy of 0.88 and AUC at 0.94. This classifier supports the feasibility of building a relatively inexpensive chip using very few sites as markers to enable detection of cancer cells based on phosphorylation assays.

These results, taken together, indicate that the phosphorylation status of proteins can be used to develop models that predict a malignant phenotype of clinical samples with very high accuracy, similar to the performance reported from mRNA expression (see Note S1) [26].

### Highly Accurate Classification of Cancer Tissue via Prediction on an Independent Validation Dataset with 16 NSCLC Samples

We applied two regression models trained from the 142 samples to an independent dataset consisting of 16 NSCLC samples. The coefficients used to integrate the phosphorylation level of proteins in the two models are shown in supplementary Table S4 and S5. At 90% sensitivity for cancer patients in the training data, the statistical model using the 20 most informative phosphorylation sites has 87.5% sensitivity on the validation samples. We repeated the analysis using the “Proliferation Genes” category from the C2 database of MSigDB. The corresponding validation sensitivity is 93.8% which is slightly better than the sensitivity obtained by the statistical model. The most informative sites used in the proliferation module classifier are derived from EGFR and SYK (Spleen Tyrosine Kinase).

Because we had no new independent normal samples to evaluate the specificity of the classifiers, we adopted a variant of a resampling approach to estimate the overall accuracy on the independent set (described in the methods section). We report the average sensitivity, specificity, accuracy, and AUC in Table S6. With seven samples left out from training data, the new classifiers showed slightly reduced sensitivity over the 16 validation cancer samples. The proliferation module classifier showed better sensitivity but lower specificity than the one based on statistically most informative markers. The estimates of accuracy for these two classifiers ranged from 84 to 88%, and the estimated AUC is 92–93%.

### Differential Phosphorylation of Cancer Associated Pathways

To gain insight into biological processes whose activity may be modulated in lung tumors, we tested 639 curated protein-sets from the canonical pathway database of MSigDB to determine if the overall phosphorylation levels of tyrosine sites in proteins from each pathway are significantly different between normal and tumor samples. We were not able to use traditional Gene Set Enrichment Analysis [25] to detect these dysregulated pathways due to the extreme sparsity of the data. Instead we associated a metaprotein representation with each pathway and computed whether the phosphorylation level of this metaprotein is correlated to changes in phenotype. This technique is a new variant of the metagene technique deployed for gene expression analysis.

In total, 181 proteins observed in this dataset were a member of at least one of the 639 protein-sets. Table 3 lists the top 15 protein-sets that display differential phosphorylation levels. The protein-set that displays the greatest change in its overall phosphorylation level when comparing normal and NSCLC tissue is the KEGG pathway “HSA05211 RENAL CELL CARCINOMA.” Of the 181 proteins considered here, 14 of them belong to this protein-set, and 9 displayed high correlation with the metaprotein phosphorylation levels. These 14 proteins are shown in Supplementary Table S7, in which a positive coefficient indicates higher phosphorylation in the tumor. Two other pathways that are especially relevant for lung cancer that have significantly different overall phosphorylation levels are “HSA05223 NON SMALL CELL LUNG CANCER” and “METPATHWAY BIOCARTA.” Five additional pathways are explicitly related to cancer: “HSA05220 CHRONIC MYELOID LEUKEMIA”, “HSA05215 PROSTATE CANCER,” “HSA05218 MELANOMA”, “HSA05213 ENDOMETRIAL CANCER” and “HSA05210 COLORECTAL CANCER.” Two pathways are generic for signaling pathways, “HSA04070 PHOSPHATIDYLINOSITOL SIGNALING” and “HSA04010 MAPK SIGNALING PATHWAY,” and the geneset with the most members was the “INTEGRIN MEDIATED CELL ADHESION GENMAPP,” with 32 observed and 28 correlated proteins.

**Table 3:** The top 15 protein-sets from MSigDB C2 database for normal/tumor classification.

*Pathway*	*# Proteins*	*# Corr Genes*	*%Var*	*FDR*
HSA05211 RENAL CELL CARCINOMA	14	9	48	0.00041
HSA04540 GAP JUNCTION	10	6	44	0.0048
HSA04662 BCR SIG PATH	14	13	33	0.016
HSA04070 PHOSPHATIDYLINOSITOL SIGNALING	12	10	32	0.016
INTEGRIN MEDIATED CELL ADHESION GENMAPP	32	28	51	0.016
HSA05220 CHRONIC MYELOID LEUKEMIA	13	7	49	0.019
HSA05120 EPITHELIAL CELL SIGNALING HP INF	17	7	81	0.019
METPATHWAY BIOCARTA	24	20	55	0.019
HSA05223 NON SMALL CELL LUNG CANCER	12	6	52	0.019
ST DIFFERENTIATION PATHWAY IN PC12 CELLS	12	8	53	0.019
HSA05215 PROSTATE CANCER	13	6	52	0.019
HSA05218 MELANOMA	13	6	52	0.019
HSA05213 ENDOMETRIAL CANCER	13	6	52	0.019
HSA05210 COLORECTAL CANCER	14	8	50	0.019
HSA04010 MAPK SIGNALING PATHWAY	15	10	80	0.019

The protein-set name is listed in column 1. The number of proteins observed from that protein-set are reported in column 2. Column 3 reports the number of proteins within the protein-set that are most responsible for the differential phosphorylation of the metaprotein. Column 4 reports the percentage of the metaprotein's variation that is differential in tumor and normal tissue. Column 5 reports the FDR *q*-value measuring the significance of the differential phosphorylation of the metaprotein associated with the pathway in cancer.

**Figure 1:**
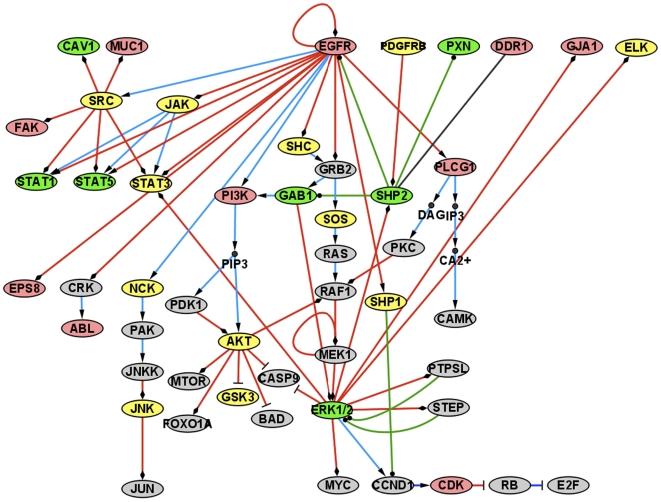
The EGFR signaling pathway. Pink indicates higher phosphorylation in tumor samples, while green indicates higher phosphorylation in normal tissue samples. Yellow nodes were observed to be phosphorylated, however did not change significantly in the two types. Gray nodes were not observed in the analysis. A red arrow (or edge) relates a kinase to its target, green edges indicate a phosphatase and its target. Blue edges indicate activation, which may not be direct. Finally, a diamond shape on the end of an edge indicates phosphorylation, while a circle indicates an inhibition of phosphorylation. Arrowheads indicate activation, which may be indirect.

Because of its pivotal role in lung cancer, we examined the EGFR pathway in greater detail. In figure 1, we map all the observed tyrosine phosphorylation events onto constituent proteins, with a model constructed from the KEGG and HPRD/NetPath databases. As expected, EGFR and many downstream proteins in the pathway are differentially phosphorylated in NSCLC samples. In total, 10 tyrosine sites are more phosphorylated in tumors (pink), 7 more phosphorylated in normal tissue (green) and 12 proteins were phosphorylated to a similar degree in both sample types. Although no clear pattern is readily evident, it is perhaps surprising to observe that the tyrosines Y186 and Y204 on ERK1 and ERK2 respectively are less phosphorylated in lung tumors. It has been observed many times that excess phosphorylation of ERK1/2 can result in cell cycle arrest, reviewed in [27], and thus the lower levels observed in tumors could result in increased cell-cycling, however, this requires additional study.

### Phosphorylation Analysis of Different Tumor Subtypes

The histopathological distinction of AD and SCC can be challenging [28], but with different treatment options available [29], an important one. We analyzed phosphorylation level differences between AD and SCC tissues at the levels of individual sites, pathways and protein-sets.

To our surprise, there are no individual sites that are statistically differentially phosphorylated in the two tumor subtypes in the current dataset. Similarly, employing the metaprotein analysis described above, we observe no significantly differentially phosphorylated pathways or protein-sets (Supplementary Table S8). The previously discussed gene models for tumor subtype classification were evaluated, with results summarized in Supplementary Table S9. The only classifier that performs reasonably well, with AUC  = 0.78, is based on EGFR features selected manually with emphasis on sites observed frequently (without consideration of cancer subtype). Assuming that all samples were diagnosed correctly, these results suggest that the differences of phosphorylation measurements between AD and SCC are small and hard to detect with pure statistical or machine learning methods. We expect that a more quantitative analysis using relative peak areas, as opposed to spectral counts, would likely perform better for this task.

### Comparison of Classifiers Based on mRNA Expression Data with Phosphotyrosine Data

For comparison, we include in the supplement a report on the accuracies of classifiers based on mRNA expression. Based on current data, it appears that phosphorylation status and mRNA expression are roughly equally informative with respect to classifying samples as tumors or normal. For distinguishing tumor subtypes, phosphotyrosines work reasonably well (AUC ∼0.78), but classifiers built with mRNA transcript levels perform significantly better (AUC ∼0.98). Neither type of data is successful at distinguishing early stage (stage I and II) from late stage tumors (stage III and IV). However, this “failure” may reflect the similar biology of the primary tumors in the two cases, which may not change significantly as metastases colonize distant sites. Interestingly, we do not observe a significant correlation between those mRNA levels that are differentially expressed, and differential tyrosine phosphorylation levels of the related protein product, highlighting the complementary nature of the information provided by activity-based measures.

## Discussion

The results presented in this paper open the door to a number of future directions in both basic and translational research. Understanding the precise role of phosphorylation measurement in regulation of signaling pathways in cancer remains an important challenge, and we primarily focused on the role of EGFR and cell proliferation pathways. We have identified a set of tyrosine residues that are differentially phosphorylated in cancerous and normal lung tissue. More than mRNA transcript or protein expression levels, the phosphorylation status of select residues are related to the functional activity of the associated gene products. This is potentially particularly important in cancers as the presence or absence of various receptor proteins may not reflect the activity of downstream signaling intermediates. This scenario could be important in at least two cases with respect to signaling pathways, one in which a receptor is present, but there is no activating ligand (or an abundance of a non-functioning ligand) to initiate downstream signaling events, or if the receptor is present, but mutated and inactive and therefore unable to transmit the binding signal.

As in any systematic genome-scale survey, the observation of unexpected results challenges the ability to derive explanatory hypotheses. Such is the case with the observation that ADH1B is the most differentially phosphorylated protein, with decreased amounts observed in tumors. Implicated in squamous aerodigestive tumors [23] where a prominent role in the metabolism of ingested ethanol is compelling, it may be the case that in lung tumors, tumor-associated hypoxia may be altering cellular carbohydrate metabolism.

The central result of the analyses presented here is the demonstration that signatures based on the differential phosphorylation of tyrosine residues exhibit robust performance at classifying cancer from normal tissue. This is true regardless if the signature is based on a large number of protein sites, a smaller number, or pathway-specific residues. This result was confirmed in both rigorous cross validation study as well as on an independent set. The classification accuracy of cancer vs. normal tissue is essentially equivalent to the results obtained by microarray experiments, even when performed on bulk-dissected tissues.

Tyrosine kinases that are hyperphosphorylated in lung tumors are hypothesized to be inappropriately activated, and can therefore be regarded as potential therapeutic targets for inhibition. In this study, we observe up to 19 tyrosine kinases that have statistically different phosphorylation levels between lung cancer and normal tissue, and nearly all of those are hyperphosphorylated in lung tumors (Supplementary Table S2).

The activation of multiple tyrosine kinases in a given cancer has been previously observed in glioblastoma multiforme [30]. In that case, combinations of tyrosine kinase inhibitors (TKIs) were necessary to substantially reduce cell viability. Rather than combinations of drugs, an alternative is to employ a multi-kinase inhibitor, such as imatinib, sorafenib and sunitinib, which are each individually capable of inhibiting multiple tyrosine kinases [31]. This led us to explore the possible existence of a multi-kinase inhibitor that targeted a large fraction of those kinases that we observed to be hyperphosphorylated in lung cancer. A comprehensive, publicly available dataset of TKI binding data [32] assayed 38 TKIs against 317 kinases. We integrated TKI binding data with our differential phosphorylation analysis (Supplementary Table S2). Of the tyrosine kinsases for which binding data was reported, and we find that, nearly all of them were bound by the TKI dasatinib with high affinity (Kd's <2 nM). This would suggest that dasatinib could potentially be a useful therapy for a selection of patients with lung cancer. More generally, our study illustrates the utility of integrating global tyrosine phosphorylation assays [14] with drug binding data to quickly arrive at potential therapeutic options, and the possibility of predicting a response to particular kinase inhibitors.

Analysis of pathway-specific total-phosphorylation levels demonstrates the specificity of the overall approach, as 7 of the top 15 protein-sets identified are explicitly defined as cancer protein-sets. Signaling pathways accounted for 4 of the remaining top 15 protein-sets, including the important examples of phosphatidylinositol and MAPK signaling pathways. Pathways downstream of the EGFR and Met receptors, with prominent roles in lung cancer biology, were similarly implicated.

While the performance of phosphotyrosine signatures was modest for the discrimination of AD and SCC, with the top performing classifier having an AUC of 0.78, mRNA transcript levels classify these subtypes with higher accuracy, and neither method distinguishes early from advanced stages of lung cancers. The latter observation may reflect a limitation of the method, or it may accurately reflect the biology of lung cancers, in that fundamentally new processes are not needed for progression, only that enough time has elapsed for the dysregulated genes and pathways to erode further into surrounding tissue and that metastases have occurred.

Future challenges for the characterization of tumors based upon phosphorylation signatures include the relative sparseness of data (especially among multiple samples), the convolution of protein levels with phosphorylation levels, and the semi-quantitative nature of spectral counting for measuring peptide abundance. However, straightforward improvements exist to address each of these limitations. It is likely that the most significant improvement would result from the use of stable isotope labels. These would allow direct comparisons among multiple samples, and increase quantitative accuracy. The fact that such excellent performance for classifying normal versus tumor tissue can already be achieved makes further improvements highly desirable. Another promise is the potential to develop signatures to guide treatments and predict patient outcomes. Kinase inhibitors are an exciting new class of treatments for cancer [33], [34], however, recent studies have emphasized that single targets of inhibition may not be sufficient to achieve a therapeutic response [30]. Combinations of kinase inhibitors, with each potentially binding to multiple targets, may be necessary to inhibit undesirable growth and proliferation signals active in neoplasms [35]. The phosphorylated proteins analyzed here represent targets of kinases that should likely be inhibited to achieve efficacy. Computational [36] or experimental [37] methods for inferring the active kinases will potentially assist in the identification of the relevant therapeutic targets in individual cases.

### Conclusions

Because the inappropriate activation of signaling pathways represents fundamental biological processes in cancer, we analyzed phosphorylation data from lung cancer and normal lung tissue to identify differences. We identified several hundred differentially phosphorylated sites in lung cancer, and developed novel computational methodology to identify pathways whose phosphorylation is dysregulated. Lastly, we demonstrated the ability to classifying lung cancers with high accuracy based on multi-variate phosphorylation signatures. The phosphorylation sites identified provide an immediate set of novel drug targets, and an analysis of the complement of sites provides a logic for the selection among potential treatments using multi-targeted kinase inhibitors or combinations of selective inhibitors.

## Materials and Methods

### Data Sources

The procedures of the experiments and the acquisition of the tyrosine-phosphorylation data were described in the previous publication [14]. The first dataset contains 151 NSCLC and 48 normal lung samples. Of the 151 tumor samples, 42 adenocarcinoma (AD) samples and 52 squamous cell carcinoma (SCC) samples with available clinical information were used in the classification analysis. Most of the 4551 observed phosphorylation sites were identified in a relatively small number of samples. For our analyses, we included those sites that were phosphorylated in at least 10% of the samples under consideration. A second dataset consisting of 16 NSCLC samples that were not originally included in [14] (and are therefore previously unpublished) were used to provide an independent validation of the predictive signature.

### Statistical Analysis for Differential Phosphorylation of Protein Sites between Sample Classes

For each site, phosphorylation levels were based on spectral counts [38]. The statistical significance of the spectral count difference between normal and tumor samples was evaluated by the Kruskal-Wallis rank sum test. This non-parametric methodology was employed because the distribution of counts did not observe a normal distribution. The *p* values were corrected for multiple hypotheses using FDR *q* values [39]. Peptides with *q* values <0.05 were considered to display statistically significant differential phosphorylation in tumor samples. We identify sites that are both “hyper-phosphorylated” in tumor samples (Tumor/Normal phosphorylation spectral count ratio >1) and ones that are “hypo-phosphorylated” in tumor samples (Tumor/Normal phosphorylation spectral count ratio <1).

### Logistic Regression Models to Predict the Sample Phenotypes

For the classification of tumor samples and normal tissue we applied a variant of a standard methodology. Five logistic regression models with different gene sets were constructed for sample phenotype classification. The first model uses all protein sites that were significantly differentially phosphorylated between two sample classes as the marker features (FDR *q* value <0.05 with Kruskal-Wallis rank sum test). Then we trained a ridge logistic regression model to fit the binary sample classes with the phosphorylation profiles of the marker protein sites by the procedures in an R package “penalized” [40]. This methodology was chosen to reduce overfitting by penalizing “model complexity” implemented in the ridge regression. An optimal penalty parameter was chosen based on the cross-validation. Models 2–4 use sets of genes that are biologically known to be related to the formation of lung cancer. Model 2 used the phosphorylated sites from “Proliferation genes” from the MSigDB C2 database [25] as the marker sites to train the logistic regression model. Models 3 and 4 used the protein sites in the EGFR pathway from two sources: a smaller core pathway protein set from BioCarta (www.biocarta.com) and a larger EGFR related protein interaction network from HPRD/NetPath [41]. Finally, we compare the performance of a minimal model, using only the top 20 performing phosphorylated tyrosines from our analysis above.

### Estimation of Model Performance for Prediction of Sample Phenotypes

To account for the potential bias due to the use of the same dataset for both model construction and model evaluation, we deployed a contextually novel strategy, based on the 0.632+ bootstrap approach [42], [43], to support the statistical validity of the relative accuracy of the classifiers reported in this paper. The approach provides a bias-corrected estimate of the prediction error by combining the bootstrap and the cross-validation, which has shown to perform better compared to the standard cross-validation approach [42], [44].

In each bootstrap instance, we created a bootstrap sample by selecting 142 observations with replacement from the original data, which on average are composed of roughly 90 unique patients. The bootstrap sample would be used as the training data for constructing logistic regression models to be used for assigning model scores. A cut-off value of the model scores which corresponds to the minimal classification errors in the training samples was taken as the classification threshold. Subsequently, we use the validation data, consisting of all the observations not in the training data, to evaluate the performance of the prediction.

In the validation data, we classify a sample with model score larger than the threshold as tumor and those below the threshold as normal tissue. We computed the overall classification accuracy as the fraction of samples whose phenotype classes were correctly predicted. We let the threshold value vary to plot a receiver operating characteristic (ROC) curve for each model using the standard methodology of increasing the threshold and documenting the corresponding error rates. The overall accuracy was summarized based on the area under the ROC curve (AUC) values. The bootstrap procedure was carried out 100 times.

Finally, bias-corrected estimates of the accuracy measures were obtained based on the 0.632+ method [42]. To assess the sampling variability of the estimated accuracy, we used the standard 95% bootstrap confidence intervals, defined by the ranges between the 2.5th and the 97.5th percentiles. The expected AUC for a random non-informative model is 0.5. A classification model was considered significantly predictive of the phenotypes only if the lower bound of the 95% confidence intervals of the AUCs is larger than the null AUC value.

### Significant Correlation of the Overall Phosphorylation of Pre-Defined Protein-Sets to Phenotypes

Due to the fact that, in general, mass spectrometric measurement of low abundance proteins is extremely sparse, we developed a metaprotein analytic approach for the analysis of pathway specific phosphorylation changes, based on pathways from MSigDB. A metaprotein is defined as a specific linear combination of selected phosphorylation sites from proteins within a curated protein-set. In the first step, for each protein-set *P*, we utilized principal component analysis on the phosphorylation profiles of the protein sites belonging to *P* in all 142 samples to define eigenvectors that reflect the variation in observed phosphorylation patterns [45]. Principal components were only considered if they explained a significant amount of the variation in metaprotein phosphorylation (>30%) and had 10 or more phosphotyrosine sites observed in the data. The metaprotein phosphorylation is defined as the sum of the phosphorylation counts of individual protein sites weighted by the coefficients in the eigenvectors corresponding to the principal components. Metaproteins with differential phosphorylation in normal tissues and tumors were identified using the Kruskal-Wallis rank sum test. Pathways with FDR *q* values less than 0.02 were considered significant. Lastly, the Pearson correlation coefficient between each phosphorylated residue and the metaprotein was computed in order to identify those proteins within the pathway that are most responsible for the observed global variation. This approach appears to be an effective methodology for identifying differentially phosphorylated pathways using proteomic data and should be broadly applicable to other types of extremely sparse data. Traditional Gene Set Enrichment Analysis [25] was not readily applicable to this type of data due to the fact that relatively few measurements per pathway were available. Combining principal component analysis, PCA, on each pathway with correlation of the derived metaproteins to phenotype produced results that are both consistent with literature as well as new findings.

### Validation on a New 16-Sample NSCLC Dataset

We compiled a set of 164 protein sites which are present both in the 142-sample and the new datasets. The most informative 20 sites on the training set were used as markers. We applied a ridge logistic regression model as described in previous sections. Multiple cutoff values of the regression scores were chosen corresponding to different sensitivity levels on the training dataset. We repeated the model construction and validation using 12 protein sites from the “Proliferation Genes” set in the C2 category of MSigDB database.

### Estimation of Specificity, Accuracy, and AUC of the Models

We randomly chose and excluded seven normal samples from the old larger dataset, and combined these normal subjects with the 16 new cancer samples to form a validation dataset. Each time we retrain a new regression model on the remainder of the training dataset with the top 20 differential sites or proliferation category proteins respectively. The feature selection was carefully performed only on training data. We repeated the resampling 100 times. The cutoff was also determined at each run, and was based on the threshold at 90% sensitivity on training samples only.

## Supporting Information

Table S1 Phosphorylation sites differentially phosphorylated between lung cancer and normal tissue.
(0.05 MB PDF)

Table S2 Differentially phosphorylated tyrosine kinases and dasatinib binding data.
(0.05 MB DOC)

Table S3 The list of protein sites in the EGFR pathway/signaling network models.
(0.05 MB DOC)

Table S4 The top 20 marker sites in the linear model.
(0.04 MB DOC)

Table S5 The 12 protein sites in the "Proliferation Genes."
(0.03 MB DOC)

Table S6 The average sensitivity, specificity, and AUC on training and validation data.
(0.03 MB DOC)

Table S7 The protein sites for the metaprotein of the C2 pathway "HSA05211 Renal Cell Carcinoma."
(0.04 MB DOC)

Table S8 The top 10 MSigDB C2 protein-sets related to AD/SCC tumor subtype distinction.
(0.03 MB DOC)

Table S9 The performances of the 5 regression models used for AD/SCC classification.
(0.03 MB DOC)

Note S1 Comparison to mRNA-based classifiers.
(0.03 MB DOC)
