# Supplementary material for: A Predictive Phosphorylation Signature of Lung Cancer
Source: PLoS One. 2009 Nov 25;4(11):e7994. doi: 10.1371/journal.pone.0007994 (PMC2777383; doi:10.1371/journal.pone.0007994)
Supplement: Table S1 — Phosphorylation sites differentially phosphorylated between lung cancer and normal tissue. (0.05 MB PDF) [file pone.0007994.s001.pdf]

Supplementary Table 1. Phosphorylation sites differentially phosphorylated between lung cancer and normal tissue.

Index	ID	FractionPH	T/N Fold Change	Ranksum_P	FDR	AUC	Symbol	Description
1	ADH1B_34	0.225	0.08	5.1E-12	1.4E-09	0.758	ADH1B	alcohol dehydrogenase IB (class I), beta polypeptide
2	CAV1_14	0.345	0.15	2.1E-11	2.3E-09	0.791	CAV1	caveolin 1, caveolae protein, 22kDa
3	TNS1_1149	0.282	0.16	2.6E-11	2.3E-09	0.77	TNS1	tensin 1
4	C11ORF52_103	0.282	0.13	4.3E-11	2.9E-09	0.768	C11orf52	chromosome 11 open reading frame 52
5	GAB1_659	0.444	0.17	3.3E-10	1.7E-08	0.791	GAB1	GRB2-associated binding protein 1
6	TNS1_1326	0.317	0.19	8.8E-10	3.9E-08	0.759	TNS1	tensin 1
7	ANXA2_29	0.408	0.2	4.4E-09	1.7E-07	0.767	ANXA2	annexin A2
8	TNS1_1404	0.437	0.13	1.1E-08	3.6E-07	0.764	TNS1	tensin 1
9	STAT1_701	0.155	0.05	1.2E-08	3.6E-07	0.684	STAT1	signal transducer and activator of transcription 1, 91kDa
10	LYN;HCK_396;410	0.493	3.87	3.5E-08	9.1E-07	0.763	LYN //// HCK	v-yes-1 Yamaguchi sarcoma viral related oncogene homolog //// hemopoietic cell kinase
11	CDC2_15	0.415	9.36	7.8E-08	1.9E-06	0.747	CDC2	cell division cycle 2, G1 to S and G2 to M
12	CDC2_15,19	0.324	13.45	1.3E-07	2.8E-06	0.725	CDC2	cell division cycle 2, G1 to S and G2 to M
13	C19ORF59_38	0.169	0.08	1.4E-07	2.8E-06	0.677	C19orf59	chromosome 19 open reading frame 59
14	SEPT2_17	0.141	0.08	1.7E-07	3.2E-06	0.662	2-Sep	septin 2
15	TNS1_1323	0.232	0.14	1.9E-07	3.3E-06	0.697	TNS1	tensin 1
16	C11ORF52_78	0.141	0.09	2.1E-07	3.4E-06	0.661	C11orf52	chromosome 11 open reading frame 52
17	TJP2_1118	0.345	0.25	3.6E-07	5.5E-06	0.719	TJP2	tight junction protein 2 (zona occludens 2)
18	PTTG1IP_174	0.549	4.03	8.6E-07	1.3E-05	0.737	PTTG1IP	pituitary tumor-transforming 1 interacting protein
19	MAPK13_182	0.725	2.72	1.0E-06	1.4E-05	0.746	MAPK13	mitogen-activated protein kinase 13
20	PIK3R2_464	0.444	4.87	1.1E-06	1.4E-05	0.727	PIK3R2	phosphoinositide-3-kinase, regulatory subunit 2 (p85 beta)
21	PTPN11_580	0.282	0.2	1.4E-06	1.7E-05	0.696	PTPN11	protein tyrosine phosphatase, non-receptor type 11 (Noonan syndrome 1)
22	MYH9_1407	0.613	2.98	1.7E-06	1.9E-05	0.729	MYH9	myosin, heavy chain 9, non-muscle
23	ACTB;ACTG1_294;294	0.627	2.71	1.7E-06	1.9E-05	0.734	ACTB //// ACTG1	actin, beta //// actin, gamma 1
24	CTNNA1_177	0.324	8	2.1E-06	2.3E-05	0.699	CTNNA1	catenin (cadherin-associated protein), alpha 1, 102kDa
25	RPS27_30	0.268	28.6	2.4E-06	2.6E-05	0.688	RPS27	ribosomal protein S27 (metallopanstimulin 1)
26	GRASP_237	0.225	0.23	2.6E-06	2.6E-05	0.676	GRASP	GRP1 (general receptor for phosphoinositides 1)-associated scaffold protein
27	CDK2;CDK3_15;15	0.289	16.09	3.3E-06	3.2E-05	0.691	CDK2 //// CDK3	cyclin-dependent kinase 2 //// cyclin-dependent kinase 3
28	APP_757;682;738	0.296	9.87	3.6E-06	3.3E-05	0.691	APP	amyloid beta (A4) precursor protein (peptidase nexin-II, Alzheimer disease)
29	GAB1_627	0.218	0.16	3.6E-06	3.3E-05	0.672	GAB1	GRB2-associated binding protein 1
30	TNS1_796	0.289	0.23	3.7E-06	3.3E-05	0.689	TNS1	tensin 1
31	SGK269_635	0.486	0.31	4.0E-06	3.4E-05	0.718	SGK269	NKF3 kinase family member
32	PXN_118;118;118	0.69	0.38	5.8E-06	4.8E-05	0.729	PXN	paxillin
33	MYH9_753	0.465	3.67	6.4E-06	5.1E-05	0.709	MYH9	myosin, heavy chain 9, non-muscle
34	HEFL_350	0.162	0.18	1.1E-05	8.3E-05	0.645	C20orf32	chromosome 20 open reading frame 32
35	ANXA2_23	0.937	0.66	1.1E-05	8.3E-05	0.719	ANXA2	annexin A2
36	PAG1_317	0.275	0.3	1.3E-05	9.9E-05	0.674	PAG1	phosphoprotein associated with glycosphingolipid microdomains 1
37	PIK3R1;PIK3R3_467;19	0.232	16.34	2.1E-05	1.5E-04	0.66	PIK3R1 //// PIK3R3	phosphoinositide-3-kinase, regulatory subunit 1 (p85 alpha) //// phosphoinositide-3-kinase, regulatory subunit 3 (p55, gamma)
38	LOC94839_707	0.317	6.41	2.2E-05	1.5E-04	0.68	---	---
39	GPRC5A_317	0.169	0.23	3.5E-05	2.4E-04	0.638	GPRC5A	G protein-coupled receptor, family C, group 5, member A
40	DYRK1A_145	0.246	10.21	5.4E-05	3.5E-04	0.656	DYRK1A	dual-specificity tyrosine-(Y)-phosphorylation regulated kinase 1A
41	TNK2_518	0.317	0.28	8.0E-05	5.1E-04	0.666	TNK2	tyrosine kinase, non-receptor, 2
42	CLTC_898	0.204	16.34	1.1E-04	7.1E-04	0.639	CLTC	clathrin, heavy chain (Hc)
43	GRLF1_1087	0.387	4.49	1.4E-04	8.3E-04	0.671	GRLF1	glucocorticoid receptor DNA binding factor 1
44	MAPK3_204	0.937	0.65	1.5E-04	8.8E-04	0.691	MAPK3	mitogen-activated protein kinase 3

Index	ID	FractionPH	T/N Fold Change	Ranksum_P	FDR	AUC	Symbol	Description
45	SLAMF1_281	0.556	2.13	1.6E-04	9.4E-04	0.682	SLAMF1	signaling lymphocytic activation molecule family member 1
46	STAT5A;STAT5B_694;69	0.951	0.61	2.0E-04	1.1E-03	0.661	STAT5A //// STAT5B	signal transducer and activator of transcription 5A //// signal transducer and activator of transcription 5B
47	VIM_116	0.246	7.15	2.5E-04	1.4E-03	0.642	VIM	vimentin
48	BAG3_457	0.176	0.16	2.6E-04	1.4E-03	0.625	BAG3	BCL2-associated athanogene 3
49	EEF1A1;EEF1A2_141;14	0.514	2.1	3.6E-04	1.9E-03	0.665	EEF1A1 //// EEF1A2	eukaryotic translation elongation factor 1 alpha 1 //// eukaryotic translation elongation factor 1 alpha 2
50	SIMILARTORGS12_28	0.408	0.39	3.7E-04	1.9E-03	0.662	---	---
51	DYRK2;DYRK4_309;286	0.486	1.87	4.0E-04	2.0E-03	0.667	DYRK2 //// DYRK4	dual-specificity tyrosine-(Y)-phosphorylation regulated kinase 2 //// dual-specificity tyrosine-(Y)-phosphorylation regulated kinase 4
52	CDC2;CDK2;CDK3_19;1	0.479	2.26	4.0E-04	2.0E-03	0.661	DC2 //// CDK2 //// CDK	cell division cycle 2, G1 to S and G2 to M //// cyclin-dependent kinase 2 //// cyclin-dependent kinase 3
53	PXN_88;88;88	0.549	0.37	6.6E-04	3.3E-03	0.663	PXN	paxillin
54	NEDD9_241	0.141	0.08	7.1E-04	3.5E-03	0.605	NEDD9	neural precursor cell expressed, developmentally down-regulated 9
55	ALB_108	0.197	7.66	8.6E-04	4.1E-03	0.618	ALB	albumin
56	HBD;HBE1;HBB;HBG1;H	0.31	0.4	1.1E-03	5.2E-03	0.635	BE1 //// HBB //// HBG1	hemoglobin, delta //// hemoglobin, epsilon 1 //// hemoglobin, beta //// hemoglobin, gamma A //// hemoglobin, gamma G
57	SYK_296	0.162	13.79	1.1E-03	5.2E-03	0.607	SYK	spleen tyrosine kinase
58	VIM_60	0.817	0.66	1.2E-03	5.2E-03	0.652	VIM	vimentin
59	EGFR_1197	0.479	2.34	1.2E-03	5.2E-03	0.65	EGFR	epidermal growth factor receptor (erythroblastic leukemia viral (v-erb-b) oncogene homolog, avian)
60	FCER1G_76	0.275	2.61	1.4E-03	5.9E-03	0.629	FCER1G	Fc fragment of IgE, high affinity I, receptor for; gamma polypeptide
61	KRT5_60	0.155	13.79	1.6E-03	7.1E-03	0.602	KRT5	keratin 5 (epidermolysis bullosa simplex, Dowling-Meara/Kobner/Weber-Cockayne types)
62	PDHA1;PDHA2_289,30	0.486	2.13	1.8E-03	7.7E-03	0.645	PDHA1 //// PDHA2	pyruvate dehydrogenase (lipoamide) alpha 1 //// pyruvate dehydrogenase (lipoamide) alpha 2
63	CD46_354	0.232	6.38	1.9E-03	7.8E-03	0.618	CD46	CD46 molecule, complement regulatory protein
64	LCP1_276	0.359	2.61	1.9E-03	7.8E-03	0.634	LCP1	lymphocyte cytosolic protein 1 (L-plastin)
65	DDR1_792,796	0.69	1.76	1.9E-03	7.8E-03	0.654	DDR1	discoidin domain receptor family, member 1
66	CDH5_685	0.197	0.27	2.2E-03	8.6E-03	0.609	CDH5	cadherin 5, type 2, VE-cadherin (vascular epithelium)
67	DDR1_792,796,797	0.148	12.77	2.3E-03	9.0E-03	0.597	DDR1	discoidin domain receptor family, member 1
68	HGS_216	0.331	2.87	2.3E-03	9.0E-03	0.629	HGS	hepatocyte growth factor-regulated tyrosine kinase substrate
69	EEF1A1;EEF1A2_29;29	0.148	11.74	2.3E-03	9.0E-03	0.596	EEF1A1 //// EEF1A2	eukaryotic translation elongation factor 1 alpha 1 //// eukaryotic translation elongation factor 1 alpha 2
70	MUC1_1203,1209	0.225	5.87	3.1E-03	1.2E-02	0.611	MUC1	mucin 1, cell surface associated
71	APLP2_750	0.141	15.32	3.3E-03	1.2E-02	0.591	APLP2	amyloid beta (A4) precursor-like protein 2
72	HSP90AB1;HSP90AB2P	0.141	9.7	3.4E-03	1.3E-02	0.591	SP90AB1 //// HSP90AB	heat shock protein 90kDa alpha (cytosolic), class B member 1 //// heat shock protein 90kDa alpha (cytosolic), class B member 2 (pseudogene)
73	ABL1;ABL2_393;412;43	0.5	2.12	3.7E-03	1.3E-02	0.638	ABL1 //// ABL2	v-abl Abelson murine leukemia viral oncogene homolog 1 //// v-abl Abelson murine leukemia viral oncogene homolog 2 (arg, Abelson-related gene)
74	SHB_333	0.134	0.26	3.7E-03	1.3E-02	0.588	SHB	Src homology 2 domain containing adaptor protein B
75	G6PD_502	0.437	2.01	3.8E-03	1.3E-02	0.632	G6PD	glucose-6-phosphate dehydrogenase
76	G6PD_506	0.169	6.64	3.8E-03	1.3E-02	0.597	G6PD	glucose-6-phosphate dehydrogenase
77	VASP_38	0.169	6.13	3.8E-03	1.3E-02	0.597	VASP	vasodilator-stimulated phosphoprotein
78	MAPK14_181	0.944	1.39	4.1E-03	1.4E-02	0.647	MAPK14	mitogen-activated protein kinase 14
79	CTNND1_228	0.239	0.34	4.1E-03	1.4E-02	0.61	CTNND1	catenin (cadherin-associated protein), delta 1

Index	ID	FractionPH	T/N Fold Change	Ranksum_P	FDR	AUC	Symbol	Description
80	NEDD9_92	0.12	0.27	4.2E-03	1.4E-02	0.583	NEDD9	neural precursor cell expressed, developmentally down-regulated 9
81	PIK3CD_524	0.134	10.21	4.8E-03	1.6E-02	0.586	PIK3CD	phosphoinositide-3-kinase, catalytic, delta polypeptide
82	GJA1_312	0.162	6.89	5.2E-03	1.7E-02	0.592	GJA1	gap junction protein, alpha 1, 43kDa
83	DLG3_673	0.169	0.35	5.2E-03	1.7E-02	0.593	DLG3	discs, large homolog 3 (neuroendocrine-dlg, Drosophila)
84	EPS8_774	0.289	3.21	5.3E-03	1.7E-02	0.613	EPS8	epidermal growth factor receptor pathway substrate 8
85	CTNND1_904	0.387	1.24	5.5E-03	1.7E-02	0.623	CTNND1	catenin (cadherin-associated protein), delta 1
86	FBP2;FBP1_216;215	0.218	3.06	6.6E-03	2.0E-02	0.6	FBP2 //// FBP1	fructose-1,6-bisphosphatase 2 //// fructose-1,6-bisphosphatase 1
87	ATP1A1_260	0.303	2.09	6.8E-03	2.1E-02	0.612	ATP1A1	ATPase, Na+/K+ transporting, alpha 1 polypeptide
88	IQGAP1_1510	0.127	9.19	6.9E-03	2.1E-02	0.58	IQGAP1	IQ motif containing GTPase activating protein 1
89	PTPN11_542	0.113	0.15	7.0E-03	2.1E-02	0.576	PTPN11	protein tyrosine phosphatase, non-receptor type 11 (Noonan syndrome 1)
90	MUC1_1203,1209,1212	0.127	20.68	7.1E-03	2.1E-02	0.58	MUC1	mucin 1, cell surface associated
91	PECAM1_713	0.528	0.31	7.2E-03	2.1E-02	0.63	PECAM1	platelet/endothelial cell adhesion molecule (CD31 antigen)
92	PRKCD_334	0.239	3.64	7.4E-03	2.1E-02	0.603	PRKCD	protein kinase C, delta
93	TENC1_359	0.754	0.69	7.7E-03	2.2E-02	0.63	TENC1	tensin like C1 domain containing phosphatase (tensin 2)
94	EPHA3;EPHA5;EPHA4_	0.183	4.26	7.8E-03	2.2E-02	0.592	IA3 //// EPHA5 //// EPH	EPH receptor A3 //// EPH receptor A5 //// EPH receptor A4
95	ICK_159	0.155	5.36	7.8E-03	2.2E-02	0.586	ICK	intestinal cell (MAK-like) kinase
96	PLCG1_977	0.232	3.78	8.0E-03	2.2E-02	0.6	PLCG1	phospholipase C, gamma 1
97	GPRC5A_347	0.634	0.62	8.4E-03	2.3E-02	0.631	GPRC5A	G protein-coupled receptor, family C, group 5, member A
98	ACTB;ACTG1;ACTA1_53	0.176	6.64	8.7E-03	2.3E-02	0.589	TB //// ACTG1 //// ACT	actin, beta //// actin, gamma 1 //// actin, alpha 1, skeletal muscle
99	ANXA2_317	0.437	0.49	8.8E-03	2.4E-02	0.62	ANXA2	annexin A2
100	DLG1_760	0.12	14.81	9.1E-03	2.4E-02	0.576	DLG1	discs, large homolog 1 (Drosophila)
101	DEFA1;DEFA3_85;85	0.204	3.83	9.4E-03	2.5E-02	0.594	DEFA1 //// DEFA3	defensin, alpha 1 //// defensin, alpha 3, neutrophil-specific
102	PTK2_925	0.113	0.27	9.5E-03	2.5E-02	0.573	PTK2	PTK2 protein tyrosine kinase 2
103	ALB_164	0.12	8.68	9.7E-03	2.5E-02	0.575	ALB	albumin
104	ACTB;ACTG1_198;198	0.38	1.82	1.1E-02	2.7E-02	0.612	ACTB //// ACTG1	actin, beta //// actin, gamma 1
105	ICK_156,159	0.176	4.09	1.1E-02	2.7E-02	0.586	ICK	intestinal cell (MAK-like) kinase
106	MAPK1_186	0.951	0.75	1.1E-02	2.8E-02	0.628	MAPK1	mitogen-activated protein kinase 1
107	EGFR_1172	0.359	1.84	1.1E-02	2.8E-02	0.61	EGFR	epidermal growth factor receptor (erythroblastic leukemia viral (v-erb-b) oncogene homolog, avian)
108	LCK_192	0.486	1.59	1.2E-02	2.8E-02	0.615	LCK	lymphocyte-specific protein tyrosine kinase
109	GAB1_406	0.352	0.44	1.2E-02	2.9E-02	0.609	GAB1	GRB2-associated binding protein 1
110	MUC1_1229	0.296	2.89	1.3E-02	3.1E-02	0.602	MUC1	mucin 1, cell surface associated
111	BCAR1_249	0.134	0.26	1.3E-02	3.1E-02	0.575	BCAR1	breast cancer anti-estrogen resistance 1
112	SLC25A5_190	0.113	7.66	1.4E-02	3.2E-02	0.569	SLC25A5	solute carrier family 25 (mitochondrial carrier; adenine nucleotide translocator), member 5
113	ERRFI1_394	0.141	8.43	1.4E-02	3.2E-02	0.576	ERRFI1	ERBB receptor feedback inhibitor 1
114	SLC25A2_138	0.183	0.36	1.4E-02	3.2E-02	0.585	SLC25A2	solute carrier family 25 (mitochondrial carrier; ornithine transporter) member 2
115	PTK2_570,576	0.141	5.87	1.4E-02	3.3E-02	0.576	PTK2	PTK2 protein tyrosine kinase 2
116	PTRF_308	0.232	0.45	1.5E-02	3.3E-02	0.593	PTRF	polymerase I and transcript release factor
117	GPRC5A_347,350	0.803	0.7	1.5E-02	3.4E-02	0.622	GPRC5A	G protein-coupled receptor, family C, group 5, member A
118	ANXA1_206	0.113	5.62	1.5E-02	3.4E-02	0.568	ANXA1	annexin A1
119	ANXA5_93	0.246	2.72	1.6E-02	3.4E-02	0.593	ANXA5	annexin A5
120	SIGLEC5_544	0.169	0.35	1.6E-02	3.5E-02	0.581	SIGLEC5	sialic acid binding Ig-like lectin 5
121	ENO1;ENO2;ENO3_43;4	0.282	2.21	1.7E-02	3.8E-02	0.597	IO1 //// ENO2 //// ENC	enolase 1, (alpha) //// enolase 2 (gamma, neuronal) //// enolase 3 (beta, muscle)
122	TKT_275	0.106	11.74	1.8E-02	3.9E-02	0.565	TKT	transketolase (Wernicke-Korsakoff syndrome)
123	TXNRD1_131	0.106	9.19	1.9E-02	4.0E-02	0.564	TXNRD1	thioredoxin reductase 1

Index	ID	FractionPH	T/N Fold Change	Ranksum_P	FDR	AUC	Symbol	Description
124	SDCBP_46	0.106	10.72	1.9E-02	4.0E-02	0.564	SDCBP	syndecan binding protein (syntenin)
125	DYRK3_369	0.268	2.26	2.0E-02	4.1E-02	0.592	DYRK3	dual-specificity tyrosine-(Y)-phosphorylation regulated kinase 3
126	MUC1_1203,1212	0.324	3.23	2.1E-02	4.4E-02	0.598	MUC1	mucin 1, cell surface associated
127	SGK223_390	0.106	0.31	2.2E-02	4.7E-02	0.562	SGK	serum/glucocorticoid regulated kinase
128	FLT1_1048,1053	0.19	2.38	2.3E-02	4.7E-02	0.58	FLT1	fms-related tyrosine kinase 1 (vascular endothelial growth factor/vascular permeability factor receptor)
129	FLNA_2387	0.232	2.89	2.4E-02	5.0E-02	0.585	FLNA	filamin A, alpha (actin binding protein 280)
130	CDC2;CDK2;CDK3_15;1	0.901	1.48	2.5E-02	5.1E-02	0.608	DC2 //// CDK2 //// CDK	cell division cycle 2, G1 to S and G2 to M //// cyclin-dependent kinase 2 //// cyclin-dependent kinase 3
131	BCAR1_128	0.218	0.37	2.6E-02	5.2E-02	0.582	BCAR1	breast cancer anti-estrogen resistance 1
132	FER_402	0.127	0.28	2.9E-02	5.7E-02	0.565	FER	fer (fps/fes related) tyrosine kinase (phosphoprotein NCP94)
133	CLTC_633	0.127	4.34	3.0E-02	5.9E-02	0.564	CLTC	clathrin, heavy chain (Hc)
134	NEDD9_166	0.127	0.3	3.0E-02	6.0E-02	0.564	NEDD9	neural precursor cell expressed, developmentally down-regulated 9
135	STAT3_704	0.972	0.75	3.3E-02	6.5E-02	0.606	STAT3	signal transducer and activator of transcription 3 (acute-phase response factor)
136	MUC1_1209	0.303	3.57	3.5E-02	6.7E-02	0.588	MUC1	mucin 1, cell surface associated
137	FLNA_1603	0.606	1.43	3.8E-02	7.3E-02	0.601	FLNA	filamin A, alpha (actin binding protein 280)
138	HIST1H2BK;H2BFS;HIST	0.12	3.83	4.1E-02	7.9E-02	0.559	// HIST2H2BF //// HIST	histone cluster 1, H2bk //// H2B histone family, member S //// histone cluster 1, H2bd //// histone cluster 1, H2bc //// histone cluster 2, H2bf //// histone cluster 1, H2bh //// histone cluster 1, H2bn //// histone cluster 1, H2bm //// histone cluster 1, H2bl
139	STAT3_705	1	0.84	4.5E-02	8.5E-02	0.601	STAT3	signal transducer and activator of transcription 3 (acute-phase response factor)
140	MAPK11_190	0.415	1.78	4.5E-02	8.5E-02	0.591	MAPK11	mitogen-activated protein kinase 11
141	TLN1_436	0.176	0.24	4.5E-02	8.5E-02	0.568	TLN1	talin 1
142	F11R_280	0.148	2.43	4.7E-02	8.7E-02	0.563	F11R	F11 receptor
143	TAGLN2;TAGLN3_191;1	0.275	0.53	4.7E-02	8.7E-02	0.579	TAGLN2 //// TAGLN3	transgelin 2 //// transgelin 3
144	HCK_208	0.359	1.7	5.0E-02	9.2E-02	0.584	HCK	hemopoietic cell kinase
145	INPPL1_986	0.239	0.51	5.1E-02	9.2E-02	0.575	INPPL1	inositol polyphosphate phosphatase-like 1
146	DDR2_736,740	0.444	1.23	5.4E-02	9.8E-02	0.589	DDR2	discoidin domain receptor family, member 2
147	AFAP1L2_413	0.901	1.19	5.5E-02	9.8E-02	0.597	AFAP1L2	actin filament associated protein 1-like 2
148	WASL_256	0.261	0.53	5.5E-02	9.9E-02	0.575	WASL	Wiskott-Aldrich syndrome-like
149	PIK3R1_452	0.19	2.86	5.7E-02	1.0E-01	0.567	PIK3R1	phosphoinositide-3-kinase, regulatory subunit 1 (p85 alpha)
150	ITGB1_783	0.246	1.7	6.4E-02	1.1E-01	0.571	ITGB1	integrin, beta 1 (fibronectin receptor, beta polypeptide, antigen CD29 includes MDF2, MSK12)
151	PTEN_174,176,177,178	0.12	0.39	7.2E-02	1.3E-01	0.552	PTEN	phosphatase and tensin homolog (mutated in multiple advanced cancers 1)
152	EPS8_525	0.106	4.6	7.3E-02	1.3E-01	0.549	EPS8	epidermal growth factor receptor pathway substrate 8
153	TLN2_1665	0.232	2.12	7.3E-02	1.3E-01	0.568	TLN2	talin 2
154	PTK2_576,577	0.401	1.53	7.3E-02	1.3E-01	0.581	PTK2	PTK2 protein tyrosine kinase 2
155	DOK1_449	0.141	0.34	7.4E-02	1.3E-01	0.555	DOK1	docking protein 1, 62kDa (downstream of tyrosine kinase 1)
156	MYH10_1415	0.296	1.94	7.6E-02	1.3E-01	0.573	MYH10	myosin, heavy chain 10, non-muscle
157	CSTB_97	0.106	3.57	7.6E-02	1.3E-01	0.549	CSTB	cystatin B (stefin B)
158	CTNND1_96	0.106	0.32	7.7E-02	1.3E-01	0.548	CTNND1	catenin (cadherin-associated protein), delta 1
159	LYN_193	0.218	1.6	7.8E-02	1.3E-01	0.565	LYN	v-yes-1 Yamaguchi sarcoma viral related oncogene homolog
160	CASKIN2_253	0.387	0.53	8.0E-02	1.3E-01	0.577	CASKIN2	CASK interacting protein 2
161	LCP1_374	0.155	2.81	8.4E-02	1.4E-01	0.556	LCP1	lymphocyte cytosolic protein 1 (L-plastin)
162	MPZL1_263	0.359	1.33	8.5E-02	1.4E-01	0.574	MPZL1	myelin protein zero-like 1
163	CYFIP1;CYFIP2_126;113	0.211	1.75	8.6E-02	1.4E-01	0.562	CYFIP1 //// CYFIP2	cytoplasmic FMR1 interacting protein 1 //// cytoplasmic FMR1 interacting protein 2
164	VIM_52,60	0.106	1.79	8.6E-02	1.4E-01	0.547	VIM	vimentin
165	NEDD9_317	0.261	0.6	8.8E-02	1.4E-01	0.567	NEDD9	neural precursor cell expressed, developmentally down-regulated 9

Index	ID	FractionPH	T/N Fold Change	Ranksum_P	FDR	AUC	Symbol	Description
166	PTPN6_564	0.204	1.82	9.0E-02	1.4E-01	0.561	PTPN6	protein tyrosine phosphatase, non-receptor type 6
167	HIPK3_359	0.824	1.16	9.2E-02	1.5E-01	0.583	HIPK3	homeodomain interacting protein kinase 3
168	LYN_472	0.275	1.69	1.0E-01	1.6E-01	0.565	LYN	v-yes-1 Yamaguchi sarcoma viral related oncogene homolog
169	ACTB;ACTG1;ACTA1;LO	0.225	1.9	1.0E-01	1.6E-01	0.561	/// ACTG1 //// ACTA1 /	actin, beta //// actin, gamma 1 //// actin, alpha 1, skeletal muscle //// ---
170	CHSY1_344	0.127	0.46	1.1E-01	1.8E-01	0.547	CHSY1	carbohydrate (chondroitin) synthase 1
171	ERRFI1_395	0.12	3.91	1.2E-01	1.8E-01	0.546	ERRFI1	ERBB receptor feedback inhibitor 1
172	MAPK8;MAPK10_185;1	0.148	0.44	1.2E-01	1.8E-01	0.549	MAPK8 //// MAPK10	mitogen-activated protein kinase 8 //// mitogen-activated protein kinase 10
173	FRK_46	0.366	1.29	1.2E-01	1.9E-01	0.568	FRK	fyn-related kinase
174	CFL1_139	0.324	1.81	1.4E-01	2.1E-01	0.563	CFL1	cofilin 1 (non-muscle)
175	VIM_52	0.669	0.76	1.4E-01	2.1E-01	0.57	VIM	vimentin
176	HBA1_24	0.859	0.84	1.5E-01	2.2E-01	0.57	HBA1	hemoglobin, alpha 1
177	PTPN18_387	0.176	1.36	1.5E-01	2.2E-01	0.549	PTPN18	protein tyrosine phosphatase, non-receptor type 18 (brain-derived)
178	SPRY1_53	0.113	0.51	1.5E-01	2.2E-01	0.541	SPRY1	sprouty homolog 1, antagonist of FGF signaling (Drosophila)
179	MUC1_1212	0.437	2.02	1.6E-01	2.4E-01	0.565	MUC1	mucin 1, cell surface associated
180	LYN_192	0.282	1.62	1.6E-01	2.4E-01	0.557	LYN	v-yes-1 Yamaguchi sarcoma viral related oncogene homolog
181	FLJ10769_85	0.514	0.59	1.6E-01	2.4E-01	0.567	FLJ10769	hypothetical protein FLJ10769
182	PDGFRA_762,768	0.261	1.58	1.6E-01	2.4E-01	0.555	PDGFRA	platelet-derived growth factor receptor, alpha polypeptide
183	CTNND1_257	0.19	0.49	1.7E-01	2.4E-01	0.548	CTNND1	catenin (cadherin-associated protein), delta 1
184	ANXA2_316	0.204	2.04	1.8E-01	2.5E-01	0.549	ANXA2	annexin A2
185	AXL_695	0.296	0.58	1.8E-01	2.6E-01	0.555	AXL	AXL receptor tyrosine kinase
186	LCP1_28	0.113	1.91	1.9E-01	2.7E-01	0.537	LCP1	lymphocyte cytosolic protein 1 (L-plastin)
187	FGR_209	0.19	0.62	1.9E-01	2.7E-01	0.546	FGR	Gardner-Rasheed feline sarcoma viral (v-fgr) oncogene homolog
188	ZNF334_165	0.155	0.55	1.9E-01	2.7E-01	0.542	ZNF334	zinc finger protein 334
189	HBD;HBB_145;145	0.254	1.57	2.1E-01	2.9E-01	0.549	HBD //// HBB	hemoglobin, delta //// hemoglobin, beta
190	INPP5D_864	0.352	1.8	2.2E-01	3.0E-01	0.554	INPP5D	inositol polyphosphate-5-phosphatase, 145kDa
191	DYRK1A;DYRK1B_319;2	0.12	0.57	2.2E-01	3.0E-01	0.536	DYRK1A //// DYRK1B	dual-specificity tyrosine-(Y)-phosphorylation regulated kinase 1A //// dual-specificity tyrosine-(Y)-phosphorylation regulated kinase 1B
192	PTK6_447	0.401	1	2.2E-01	3.0E-01	0.445	PTK6	PTK6 protein tyrosine kinase 6
193	LCK;FYN;YES1;SRC_394	0.789	0.62	2.2E-01	3.0E-01	0.438	//// FYN //// YES1 ////	lymphocyte-specific protein tyrosine kinase //// FYN oncogene related to SRC, FGR, YES //// v-yes-1 Yamaguchi sarcoma viral oncogene homolog 1 //// v-src sarcoma (Schmidt-Ruppin A-2) viral oncogene homolog (avian)
194	TLN1_70	0.648	1.23	2.2E-01	3.0E-01	0.558	TLN1	talin 1
195	TYROBP_91	0.268	1.05	2.3E-01	3.0E-01	0.548	TYROBP	TYRO protein tyrosine kinase binding protein
196	SFTPC_16	0.324	0.78	2.3E-01	3.1E-01	0.551	SFTPC	surfactant, pulmonary-associated protein C
197	DOK2_299	0.176	0.65	2.4E-01	3.2E-01	0.54	DOK2	docking protein 2, 56kDa
198	MAPK9_185;185	0.331	0.69	2.6E-01	3.4E-01	0.548	MAPK9	mitogen-activated protein kinase 9
199	HGS_132	0.127	2.35	2.8E-01	3.7E-01	0.532	HGS	hepatocyte growth factor-regulated tyrosine kinase substrate
200	PTK2_577	0.549	1.12	2.8E-01	3.7E-01	0.552	PTK2	PTK2 protein tyrosine kinase 2
201	HBB_130	0.268	1.32	2.9E-01	3.8E-01	0.542	HBB	hemoglobin, beta
202	FAM62A_822	0.901	0.89	2.9E-01	3.8E-01	0.549	FAM62A	family with sequence similarity 62 (C2 domain containing), member A
203	MUC1_1203	0.268	2.04	3.1E-01	4.1E-01	0.54	MUC1	mucin 1, cell surface associated
204	LSR_503	0.12	1.91	3.3E-01	4.2E-01	0.528	LSR	lipolysis stimulated lipoprotein receptor
205	RPSA;LOC387867_138;	0.254	1.53	3.4E-01	4.3E-01	0.537	RPSA //// LOC387867	ribosomal protein SA //// similar to 40S ribosomal protein SA (p40) (34/67 kDa laminin receptor) (Colon carcinoma laminin-binding protein) (NEM/1CHD4) (Multidrug resistance-associated protein MGr1-Ag)
206	PIK3R1_467	0.824	1.09	3.4E-01	4.3E-01	0.546	PIK3R1	phosphoinositide-3-kinase, regulatory subunit 1 (p85 alpha)

Index	ID	FractionPH	T/N Fold Change	Ranksum_P	FDR	AUC	Symbol	Description
207	FYN;YES1_214;222	0.12	1.66	3.4E-01	4.3E-01	0.527	FYN //// YES1	FYN oncogene related to SRC, FGR, YES //// v-yes-1 Yamaguchi sarcoma viral oncogene homolog 1
208	FYB_571	0.239	0.56	3.4E-01	4.3E-01	0.536	FYB	FYN binding protein (FYB-120/130)
209	GPRC5A_350	0.521	0.69	3.5E-01	4.4E-01	0.544	GPRC5A	G protein-coupled receptor, family C, group 5, member A
210	HIPK1;HIPK2_352;361	0.824	1	3.5E-01	4.5E-01	0.547	HIPK1 //// HIPK2	homeodomain interacting protein kinase 1 //// homeodomain interacting protein kinase 2
211	PTK2_576	0.894	0.81	3.8E-01	4.7E-01	0.545	PTK2	PTK2 protein tyrosine kinase 2
212	FYN;YES1;FGR_185;193	0.141	1.45	3.8E-01	4.8E-01	0.527	FYN //// YES1 //// FGR	FYN oncogene related to SRC, FGR, YES //// v-yes-1 Yamaguchi sarcoma viral oncogene homolog 1 //// Gardner-Rasheed feline sarcoma viral (v-fgr) oncogene homolog
213	TNS1_339,366	0.331	0.61	3.9E-01	4.8E-01	0.537	TNS1	tensin 1
214	LPP_317	0.155	0.65	4.1E-01	5.1E-01	0.526	LPP	LIM domain containing preferred translocation partner in lipoma
215	BCAR1_234	0.239	0.62	4.2E-01	5.2E-01	0.531	BCAR1	breast cancer anti-estrogen resistance 1
216	TLN1_1116	0.113	1.53	4.3E-01	5.3E-01	0.522	TLN1	talin 1
217	KIAA1217_239	0.521	1.06	4.5E-01	5.4E-01	0.535	KIAA1217	KIAA1217
218	LDHA_238	0.134	1.53	4.5E-01	5.4E-01	0.523	LDHA	lactate dehydrogenase A
219	DYRK1A;DYRK1B_321;2	0.979	1.17	4.6E-01	5.5E-01	0.538	DYRK1A //// DYRK1B	dual-specificity tyrosine-(Y)-phosphorylation regulated kinase 1A //// dual-specificity tyrosine-(Y)-phosphorylation regulated kinase 1B
220	GPRC5A_320	0.134	1.45	4.7E-01	5.6E-01	0.522	GPRC5A	G protein-coupled receptor, family C, group 5, member A
221	MAPK7_220;221	0.43	1.13	4.7E-01	5.6E-01	0.533	MAPK7	mitogen-activated protein kinase 7
222	PTK2B_579,580	0.176	0.77	4.7E-01	5.6E-01	0.524	PTK2B	PTK2B protein tyrosine kinase 2 beta
223	TNS1_366	0.232	0.87	4.8E-01	5.7E-01	0.527	TNS1	tensin 1
224	PTPRA_798;789	0.359	0.65	4.9E-01	5.7E-01	0.531	PTPRA	protein tyrosine phosphatase, receptor type, A
225	PECR_179	0.155	1.31	4.9E-01	5.8E-01	0.522	PECR	peroxisomal trans-2-enoyl-CoA reductase
226	PRPF4B_849	0.979	1.05	5.2E-01	6.1E-01	0.533	PRPF4B	PRP4 pre-mRNA processing factor 4 homolog B (yeast)
227	PTPN11_62	0.683	1.18	5.4E-01	6.3E-01	0.529	PTPN11	protein tyrosine phosphatase, non-receptor type 11 (Noonan syndrome 1)
228	TYK2_292	0.197	1.12	5.7E-01	6.5E-01	0.52	TYK2	tyrosine kinase 2
229	FMO4_432	0.106	0.66	5.7E-01	6.5E-01	0.516	FMO4	flavin containing monooxygenase 4
230	PIK3R1_580	0.345	1.26	5.7E-01	6.5E-01	0.524	PIK3R1	phosphoinositide-3-kinase, regulatory subunit 1 (p85 alpha)
231	TNS1_903	0.239	0.45	5.7E-01	6.5E-01	0.522	TNS1	tensin 1
232	GRLF1_1105	0.972	0.9	5.7E-01	6.5E-01	0.528	GRLF1	glucocorticoid receptor DNA binding factor 1
233	RA70_197	0.211	0.69	5.9E-01	6.6E-01	0.52	SKAP2	src kinase associated phosphoprotein 2
234	RIN1_36	0.324	1.13	5.9E-01	6.7E-01	0.523	RIN1	Ras and Rab interactor 1
235	MAPK12_185	0.585	0.93	6.0E-01	6.8E-01	0.474	MAPK12	mitogen-activated protein kinase 12
236	TLN1_26	0.268	0.92	6.1E-01	6.8E-01	0.521	TLN1	talin 1
237	ANK3_533	0.19	1.23	6.1E-01	6.8E-01	0.518	ANK3	ankyrin 3, node of Ranvier (ankyrin G)
238	PRKCD_313	0.577	1.07	6.3E-01	7.0E-01	0.523	PRKCD	protein kinase C, delta
239	ANXA2_315	0.338	1.12	6.5E-01	7.2E-01	0.519	ANXA2	annexin A2
240	CLDN5_212,217	0.141	1.36	6.7E-01	7.4E-01	0.513	CLDN5	claudin 5 (transmembrane protein deleted in velocardiofacial syndrome)
241	PGAM2;PGAM1_92;91	0.739	1.09	6.7E-01	7.4E-01	0.521	PGAM2 //// PGAM1	phosphoglycerate mutase 2 (muscle) //// phosphoglycerate mutase 1 (brain)
242	PDHA1;PDHA2_301;29	0.641	0.76	7.0E-01	7.6E-01	0.519	PDHA1 //// PDHA2	pyruvate dehydrogenase (lipoamide) alpha 1 //// pyruvate dehydrogenase (lipoamide) alpha 2
243	SYK_323	0.289	1.12	7.2E-01	7.8E-01	0.515	SYK	spleen tyrosine kinase
244	MUC1_1209,1212	0.366	2.39	7.2E-01	7.8E-01	0.516	MUC1	mucin 1, cell surface associated
245	FGR_208	0.197	1.33	7.5E-01	8.1E-01	0.511	FGR	Gardner-Rasheed feline sarcoma viral (v-fgr) oncogene homolog
246	FCER1G_65	0.655	1.03	7.9E-01	8.4E-01	0.513	FCER1G	Fc fragment of IgE, high affinity I, receptor for; gamma polypeptide
247	EGFR_1092	0.176	1.21	7.9E-01	8.5E-01	0.509	EGFR	epidermal growth factor receptor (erythroblastic leukemia viral (v-erb-b) oncogene homolog, avian)

Index	ID	FractionPH	T/N Fold Change	Ranksum_P	FDR	AUC	Symbol	Description
248	TAGLN_192	0.282	0.71	8.2E-01	8.7E-01	0.509	TAGLN	transgelin
249	TGM2_369	0.458	0.83	8.3E-01	8.8E-01	0.51	TGM2	transglutaminase 2 (C polypeptide, protein-glutamine-gamma-glutamyltransferase)
250	VCL_821;821	0.901	1.03	8.5E-01	9.0E-01	0.509	VCL	vinculin
251	DDR1_792	0.113	0.94	8.5E-01	9.0E-01	0.495	DDR1	discoidin domain receptor family, member 1
252	GSK3A;GSK3B_279;216	1	1.03	8.6E-01	9.0E-01	0.491	GSK3A //// GSK3B	glycogen synthase kinase 3 alpha //// glycogen synthase kinase 3 beta
253	HIST1H4A;HIST1H4F_8	0.282	0.94	8.7E-01	9.0E-01	0.493	HIST1H4A //// HIST1H4	histone cluster 1, H4a //// histone cluster 1, H4f
254	VCL_691;691	0.465	1.05	8.7E-01	9.0E-01	0.492	VCL	vinculin
255	FGR_412	0.12	0.88	8.8E-01	9.1E-01	0.504	FGR	Gardner-Rasheed feline sarcoma viral (v-fgr) oncogene homolog
256	SHC1_427;317	0.718	1.06	9.0E-01	9.3E-01	0.506	SHC1	SHC (Src homology 2 domain containing) transforming protein 1
257	TPM3;TPM4_162;126;1	0.162	0.96	9.1E-01	9.4E-01	0.504	TPM3 //// TPM4	tropomyosin 3 //// tropomyosin 4
258	CALM1_99	0.739	1.01	9.2E-01	9.4E-01	0.505	CALM1	calmodulin 1 (phosphorylase kinase, delta)
259	INPP5D_1021	0.204	0.93	9.3E-01	9.5E-01	0.503	INPP5D	inositol polyphosphate-5-phosphatase, 145kDa
260	PTK2_397	0.204	0.83	9.4E-01	9.5E-01	0.503	PTK2	PTK2 protein tyrosine kinase 2
261	FYN;YES1_213;221	0.676	0.93	9.4E-01	9.6E-01	0.503	FYN //// YES1	FYN oncogene related to SRC, FGR, YES //// v-yes-1 Yamaguchi sarcoma viral oncogene homolog 1
262	ANXA2_237	0.373	1.13	9.5E-01	9.6E-01	0.503	ANXA2	annexin A2
263	FLJ32810_373	0.232	1.05	9.5E-01	9.6E-01	0.502	FLJ32810	hypothetical protein FLJ32810
264	S100A10_24	0.57	0.98	9.7E-01	9.7E-01	0.498	S100A10	S100 calcium binding protein A10
