# Supplementary material for: A Predictive Phosphorylation Signature of Lung Cancer
Source: PLoS One. 2009 Nov 25;4(11):e7994. doi: 10.1371/journal.pone.0007994 (PMC2777383; doi:10.1371/journal.pone.0007994)
Supplement: Table S2 — Differentially phosphorylated tyrosine kinases and dasatinib binding data. (0.05 MB DOC) [file pone.0007994.s002.doc]

**Table S2.** Differentially phosphorylated tyrosine kinases in lung cancer and normal tissue, and dasatinib binding data [1]. Genes symbols are listed in column 1. Column 2 are the FDR *q*-values. Inspection of columns 2 and 3 reveal that of the TKs that are differentially phosphorylated, nearly all are hyperphosphorylated in tumors. Column 5 reports binding constants (nM) for those proteins that satisfy Kd < 2 nM. Inspection of columns 4 and 5 reveal that for those TKs with reported binding data, nearly all are bound tightly by Dasatinib. Cells are merged when the identified peptides are identical in both proteins, and thus either protein could have been the source. Binding data was evaluated for each protein individually. One protein, PTK2 (FAK), had residues that were hyperphosphorylated (Y570, Y576) and hypophosphorylated (Y925) in tumors.

| **Tyrosine Kinase** | **Statistically Different Phosphorylation** | **Hyperphos- phorylated in Lung Cancer** | **Binding Data Available^[1]^** | **Dasatinib Binding**  **Kd < 2 nM** |
| --- | --- | --- | --- | --- |
| **ABL1** | 0.01 | ✓ | ✓ | 0.53 |
| **ABL2** |  |  | ✓ | 0.17 |
| **DDR1** | 0.01 | ✓ | ✓ | 0.69 |
| **EPHA3** | 0.02 | ✓ | ✓ | 0.093 |
| **EPHA4** |  |  | ✓ | 1.2 |
| **EPHA5** |  |  | ✓ | 0.24 |
| **LCK** | 0.03 | ✓ | ✓ | 0.2 |
| **LYN** | 9.1E-07 | ✓ | ✓ | 0.57 |
| **HCK** |  |  | ✓ | 0.35 |
| **EGFR** | 0.01 | ✓ | ✓ |  |
| **FLT1** | 0.05 | ✓ | ✓ |  |
| **SYK** | 0.01 | ✓ | ✓ |  |
| **DYRK1A** | 3.5E-04 | ✓ |  |  |
| **DYRK3** | 0.04 | ✓ |  |  |
| **DYRK2** | 2.0E-03 | ✓ |  |  |
| **DYRK4** |  |  |  |  |
| **PTK2** | 0.02 | +/- |  |  |
| **SGK269** | 3.4E-05 |  |  |  |
| **TNK2** | 5.1E-04 |  | ✓ |  |
