# Supplementary material for: A Predictive Phosphorylation Signature of Lung Cancer
Source: PLoS One. 2009 Nov 25;4(11):e7994. doi: 10.1371/journal.pone.0007994 (PMC2777383; doi:10.1371/journal.pone.0007994)
Supplement: Table S3 — The list of protein sites in the EGFR pathway/signaling network models. (0.05 MB DOC) [file pone.0007994.s003.doc]

**Table S3.** The list of protein sites in the EGFR pathway/signaling network. Two ridge regularized regression models were built with all EGFR signaling network proteins or only with the core pathway proteins. The significances of the protein sites in the model stand for the significance of the Pearson correlation coefficients between the integrated regression model scores to the individual phosphorylation profiles across all the samples in the dataset.

| **Protein sites** | **Significant in the expanded model** | **Core EGFR pathway protein (BioCarta)** | **Significant in the core EGFR model** |
| --- | --- | --- | --- |
| EGFR_1172 | Yes | X | Yes |
| EGFR_1197 | Yes | X | Yes |
| MAPK3_204 | Yes | X | Yes |
| PIK3R1_452 | Yes | X | Yes |
| PLCG1_977 | Yes | X | Yes |
| STAT1_701 | Yes | X | Yes |
| BCAR1_249 | Yes |  |  |
| CAV1_14 | Yes |  |  |
| CTNND1_228 | Yes |  |  |
| EPS8_774 | Yes |  |  |
| ERRFI1_394 | Yes |  |  |
| ERRFI1_395 | Yes |  |  |
| GAB1_406 | Yes |  |  |
| GAB1_627 | Yes |  |  |
| GAB1_659 | Yes |  |  |
| GJA1_312 | Yes |  |  |
| MAPK14_181 | Yes |  |  |
| PIK3CD_524 | Yes |  |  |
| PIK3R1;PIK3R3_467;199 | Yes |  |  |
| PIK3R2_464 | Yes |  |  |
| PTPN11_542 | Yes |  |  |
| PTPN11_580 | Yes |  |  |
| PXN_118;118;118 | Yes |  |  |
| PXN_88;88;88 | Yes |  |  |
| STAT5A;STAT5B_694;699 | Yes |  |  |
| TNK2_518 | Yes |  |  |
| STAT3_704 | No | X | Yes |
| STAT3_705 | No | X | Yes |
