# Supplementary material for: A Predictive Phosphorylation Signature of Lung Cancer
Source: PLoS One. 2009 Nov 25;4(11):e7994. doi: 10.1371/journal.pone.0007994 (PMC2777383; doi:10.1371/journal.pone.0007994)
Supplement: Table S4 — The top 20 marker sites in the linear model. (0.04 MB DOC) [file pone.0007994.s004.doc]

**Table S4.** The top 20 marker sites in a linear model trained on the original 142-sample dataset with an L2 penalized logistic regression model. The magnitude of the coefficients indicates the optimized weight of the protein in the classifier. Negative coefficients indicate hypophosphorylated in tumors.

| **Marker sites** | **Coefficients** |
| --- | --- |
| ADH1B_34 | -0.622 |
| CAV1_14 | -0.237 |
| TNS1_1149 | 0.084 |
| C11ORF52_103 | -0.379 |
| GAB1_659 | -0.07 |
| TNS1_1326 | -0.509 |
| ANXA2_29 | -0.79 |
| TNS1_1404 | -0.255 |
| STAT1_701 | -1.244 |
| LYN;HCK_396;410 | 0.811 |
| CDC2_15 | 0.319 |
| C19ORF59_38 | 0.321 |
| SEPT2_17 | 0.244 |
| TNS1_1323 | 0.028 |
| C11ORF52_78 | -0.149 |
| TJP2_1118 | -0.452 |
| PTTG1IP_174 | 0.223 |
| MAPK13_182 | 0.56 |
| PIK3R2_464 | 0.726 |
| MYH9_1407 | 0.555 |
