# Supplementary material for: A Predictive Phosphorylation Signature of Lung Cancer
Source: PLoS One. 2009 Nov 25;4(11):e7994. doi: 10.1371/journal.pone.0007994 (PMC2777383; doi:10.1371/journal.pone.0007994)
Supplement: Table S5 — The 12 protein sites in the "Proliferation Genes." (0.03 MB DOC) [file pone.0007994.s005.doc]

**Table S5.** The 12 protein sites in the “Proliferation Genes” set from the C2 category of MsigDB database.

| **Marker sites** | **Coefficients** |
| --- | --- |
| DLG3_673 | -1.628 |
| EGFR_1172 | 1.653 |
| EGFR_1197 | 1.148 |
| EPS8_525 | 0.032 |
| EPS8_774 | 0.726 |
| FRK_46 | 0.374 |
| GAB1_406 | -0.001 |
| GAB1_627 | -0.468 |
| GAB1_659 | -1.309 |
| HGS_216 | 1.36 |
| SYK_323 | 0.201 |
| SYK_296 | 2.363 |
