# Supplementary material for: A Predictive Phosphorylation Signature of Lung Cancer
Source: PLoS One. 2009 Nov 25;4(11):e7994. doi: 10.1371/journal.pone.0007994 (PMC2777383; doi:10.1371/journal.pone.0007994)
Supplement: Table S6 — The average sensitivity, specificity, and AUC on training and validation data. (0.03 MB DOC) [file pone.0007994.s006.doc]

**Table S6.** The average sensitivity, specificity, and AUC on training and validation data across 100 resampling analyses. At each run, seven normal samples were randomly chosen from the old 142-sample data and used together with the 16 new cancer samples to estimate specificity and AUC. The score threshold is the cutoff of 90% sensitivity on the training data.

| **Models** | **Training Specificity** | **AUC** | **Validation sensitivity** | **Specificity** | **Accuracy** | **AUC** |
| --- | --- | --- | --- | --- | --- | --- |
| Top 20 sites | 95.4 | 98.8 | 84.1 | 93.9 | 84.6 | 92.2 |
| Proliferation Genes (12 sites) | 83.5 | 95.5 | 93.4 | 75.1 | 87.8 | 93 |
