# Supplementary material for: A Predictive Phosphorylation Signature of Lung Cancer
Source: PLoS One. 2009 Nov 25;4(11):e7994. doi: 10.1371/journal.pone.0007994 (PMC2777383; doi:10.1371/journal.pone.0007994)
Supplement: Table S7 — The protein sites for the metaprotein of the C2 pathway "HSA05211 Renal Cell Carcinoma." (0.04 MB DOC) [file pone.0007994.s007.doc]

**Table S7.** The protein sites for the metaprotein of the C2 pathway “HSA05211 Renal Cell Carcinoma**”** for normal / tumor classification. A positive coefficient in the table indicates high phosphorylation in tumor samples.

| **Index** | **Protein sites** | **FDR** | **Coeff** |
| --- | --- | --- | --- |
| 1 | MAPK3_204 | < 1e-9 | -0.582 |
| 2 | MAPK1_186 | < 1e-9 | -0.542 |
| 3 | GAB1_659 | < 1e-9 | -0.422 |
| 4 | GAB1_406 | < 1e-9 | -0.232 |
| 5 | PTPN11_580 | < 1e-9 | -0.193 |
| 6 | GAB1_627 | < 1e-9 | -0.178 |
| 7 | PIK3R1_467 | 4.30E-09 | -0.154 |
| 8 | PIK3R1_580 | 1.60E-08 | -0.163 |
| 9 | PTPN11_542 | 0.004 | -0.075 |
| 10 | PIK3R2_464 | 0.014 | 0.083 |
| 11 | PIK3R1;PIK3R3_467;199 | 0.023 | 0.03 |
| 12 | PTPN11_62 | 0.119 | -0.048 |
| 13 | PIK3CD_524 | 0.617 | -0.007 |
| 14 | PIK3R1_452 | 0.857 | -0.003 |
