# Supplementary material for: A Predictive Phosphorylation Signature of Lung Cancer
Source: PLoS One. 2009 Nov 25;4(11):e7994. doi: 10.1371/journal.pone.0007994 (PMC2777383; doi:10.1371/journal.pone.0007994)
Supplement: Table S8 — The top 10 MSigDB C2 protein-sets related to AD/SCC tumor subtype distinction. (0.03 MB DOC) [file pone.0007994.s008.doc]

**Table S8.** The top 10 MSigDB C2 protein-sets related to AD/SCC tumor subtype distinction

| **Pathway** | **# genes** | **# corr genes** | **%Var** | **FDR** |
| --- | --- | --- | --- | --- |
| BREAST CANCER ESTROGEN SIGNALING | 13 | 9 | 79 | 0.126 |
| CXCR4PATHWAY | 17 | 14 | 64 | 0.126 |
| MCALPAINPATHWAY | 18 | 12 | 62 | 0.126 |
| METPATHWAY | 22 | 18 | 57 | 0.126 |
| HSA01430 CELL COMMUNICATION | 12 | 5 | 37 | 0.126 |
| ECMPATHWAY | 19 | 14 | 61 | 0.126 |
| VEGFPATHWAY | 15 | 9 | 67 | 0.126 |
| HSA04810 REGULATION OF ACTIN CYTOSKELETON | 37 | 20 | 47 | 0.126 |
| INTEGRINPATHWAY | 21 | 16 | 59 | 0.126 |
| HSA04510 FOCAL ADHESION | 45 | 27 | 45 | 0.126 |
