# Supplementary material for: A Predictive Phosphorylation Signature of Lung Cancer
Source: PLoS One. 2009 Nov 25;4(11):e7994. doi: 10.1371/journal.pone.0007994 (PMC2777383; doi:10.1371/journal.pone.0007994)
Supplement: Table S9 — The performances of the 5 regression models used for AD/SCC classification. (0.03 MB DOC) [file pone.0007994.s009.doc]

**Table S9.**

The performances of the 5 regression models used for AD/SCC classification. Shown in the table are the mean classification accuracy and AUC across the 100 bootstraps. The 95% bootstrap confidence intervals of the accuracy and AUC are in the parentheses.

| **Marker sites used in the regression models** | **Classification Accuracy  (95% C.I.)** | **AUC  (95% C.I.)** | **Average no. of marker sites** |
| --- | --- | --- | --- |
| Differentially phosphorylated sites | 0.536  (0.406~0.670) | 0.550  (0.392~0.749) | 29 |
| Proliferation category | 0.577  (0.42~0.712) | 0.604  (0.433~0.773) | 20 |
| EGFR pathway from BioCarta | 0.570  (0.419~0.693) | 0.565  (0.410~0.683) | 11 |
| EGFR signaling network from HPRD | 0.608  (0.444~0.798) | 0.643  (0.465~0.824) | 47 |
| Nine protein sites in EGFR pathway with high  sample phosphorylation fractions* | 0.678  (0.542~0.775) | 0.781  (0.635~0.874) | 7 |
| Top 20 differentially phosphorylated | 0.540  (0.385~0.678) | 0.543  (0.378~0.717) | 20 |

* GAB1_627, PIK3R1_467, PIK3R1_556, PIK3R1_580, PIK3R2;PIK3R1;PIK3R3_464;467;199, PTPN11_62, PXN_88;88;88, and WASL_256.
