# Supplementary material for: A Predictive Phosphorylation Signature of Lung Cancer
Source: PLoS One. 2009 Nov 25;4(11):e7994. doi: 10.1371/journal.pone.0007994 (PMC2777383; doi:10.1371/journal.pone.0007994)
Supplement: Note S1 — Comparison to mRNA-based classifiers. (0.03 MB DOC) [file pone.0007994.s010.doc]

**SUPPLEMENTARY NOTE S1**

**Comparison to mRNA-based classifiers**

We compared the capability of phosphotyrosine profiles and mRNA expression profiles in classifying different classes of normal or lung cancer samples and different subtypes of lung cancer. We used a published NSCLC dataset [1] to construct and validate the mRNA expression classification models. A regularized logistic regression classifier based on mRNA levels also performed very well in separating the normal lung from cancer samples (accuracy ~ 0.965, AUC ~ 0.974). In separating AD from SCC, mRNA-based classifiers performed better than those using phosphopeptide levels (accuracy ~ 0.952, AUC ~ 0.977). However, the models based on mRNA profiles did not successfully predict the tumor stages (the AUC ~ 0.593, no statistical difference from those based on phosphorylation profiles).

**References**

1. Bhattacharjee A, Richards WG, Staunton J, Li C, Monti S, Vasa P, Ladd C, Beheshti J, Bueno R, Gillette M, et al: **Classification of human lung carcinomas by mRNA expression profiling reveals distinct adenocarcinoma subclasses.** *Proc Natl Acad Sci U S A* 2001, **98:**13790-13795.
